# Development of a Decellularized Porcine Esophageal Matrix for Potential Applications in Cancer Modeling

**DOI:** 10.3390/cells10051055

**Published:** 2021-04-29

**Authors:** Hersh Chaitin, Michael L. Lu, Michael B. Wallace, Yunqing Kang

**Affiliations:** 1 Department of Biological Science, College of Science, Florida Atlantic University, Boca Raton, FL 33431, USA; hchaitin2012@health.fau.edu; 2 Department of Biomedical Science, College of Medicine, Florida Atlantic University, Boca Raton, FL 33431, USA; MLU3@health.fau.edu; 3 Department of Gastroenterology and Hepatology, Mayo Clinic, Jacksonville, FL 32224, USA; Wallace.Michael@mayo.edu; 4 Department of Ocean and Mechanical Engineering, College of Computer Science and Engineering, Florida Atlantic University, Boca Raton, FL 33431, USA; 5 Faculty of Integrative Biology PhD Program, College of Science, Florida Atlantic University, Boca Raton, FL 33431, USA

**Keywords:** decellularization, decellularized extracellular matrix, esophageal cancer, tumor model

## Abstract

Many decellularized extracellular matrix-derived whole organs have been widely used in studies of tissue engineering and cancer models. However, decellularizing porcine esophagus to obtain decellularized esophageal matrix (DEM) for potential biomedical applications has not been widely investigated. In this study a modified decellularization protocol was employed to prepare a porcine esophageal DEM for the study of cancer cell growth. The cellular removal and retention of matrix components in the porcine DEM were fully characterized. The microstructure of the DEM was observed using scanning electronic microscopy. Human esophageal squamous cell carcinoma (ESCC) and human primary esophageal fibroblast cells (FBCs) were seeded in the DEM to observe their growth. Results show that the decellularization process did not cause significant loss of mechanical properties and that blood ducts and lymphatic vessels in the submucosa layer were also preserved. ESCC and FBCs grew on the DEM well and the matrix did not show any toxicity to cells. When FBS and ESCC were cocultured on the matrix, they secreted more periostin, a protein that supports cell adhesion on matrix. This study shows that the modified decellularization protocol can effectively remove the cell materials and maintain the microstructure of the porcine esophageal matrix, which has the potential application of studying cell growth and migration for esophageal cancer models.

## 1. Introduction

Esophageal cancer is the sixth leading cause of cancer-related deaths worldwide [1] with a very limited number of effective treatments. Limited progress in treating this deadly cancer is due, in part, to the lack of a physiologically relevant model, as an in vitro esophagus cancer model is crucial to developing new therapeutic strategies for treatment.

Currently, pre-clinical research on esophageal cancer phenotype, aggressiveness, metastasis, and drug discovery is commonly performed in two-dimensional (2D) in vitro cancer cell culture models. New tissue engineering strategies are also being used to develop esophageal cancer models to replicate the tumor-specific cellular and matrix microenvironments. Such efforts being made using tissue-engineering concepts are growing cancer cells in synthetic porous polymer scaffolds or nature collagen/Matrigel hydrogels to form artificial tumor tissue. These models have the potential to overcome the limitations of 2D monolayer culture [2,3,4,5], as they help mimic cancer growth, progression, and metastasis by providing closer matrix environments [6]. However, those synthetic polymer scaffolds, such as poly(lactic acid) (PLA) [7] and poly(lactic-co-glycolic acid) (PLGA) scaffolds [8], or natural collagen/Matrigel [9,10], do not fully reflect the composition of the extracellular matrix (ECM) of the human esophagus [7]. Previous experiments have investigated decellularized rat esophagus for medical applications in humans [11,12,13]. Unfortunately, the rat esophagus does not contain submucosal glands in their esophagus, such as those found in pigs or humans [14], therefore any attempt to recapitulate the human esophagus in the rat model would not contain all relevant anatomical attributes found in humans.

The purpose of this paper is to produce a porcine DEM as a valuable scaffold for the purpose of observing how a porcine DEM supports cells to adhere to the matrix and subsequently migrate into the submucosa. It has been previously reported that esophageal squamous cell carcinoma (ESCC) can have multiple lymph node involvement in as early as stage II, prior to the primary tumor invading past the lamina propria. The mechanisms that facilitate this early cancer cell migration are largely unknown, but have been reported in peer-reviewed publications based on human pathology reports as “skipped metastasis” [15,16]. Therefore, the development of a biomimetic porcine DEM scaffold could offer deeper insight into the effect of a biomimetic matrix on cell growth behaviors of cancer cells in the ECM. The esophageal ECM consists of distinct structural layers: the mucosa (submucosa) and the muscularis externa, which coordinates the transportation of food bolus. The innermost layer of the esophageal wall is the stratified, non-keratinized squamous epithelium with its basement membrane. The submucosa is a collagen-based matrix abundant with networks of ducts and vessels. The muscularis externa forms the muscular wall of the esophageal tract and muscle cell type changes along the length of muscularis externa to help the movement of food. In order to retain such complex, native ECM, a new modified decellularization technique needs to be developed to produce a biomimetic-supporting matrix and structures for cancer cell adhesion and growth.

Decellularization methods have been developed to produce a decellularized matrix that preserves native ECM, surface topography, and biomechanical properties of the native tissue that can support successful recellularization and function [17]. Porcine esophageal ECM has similar size and tubular structure to human esophagus. It is the closest biological equivalent to human esophageal tissue as a model substrate for cancer cell behavior. The matrix is heterogenous in layering, composed of human compatible constituent components, and has a comparable submucosal stiffness to native human tissue [18]. However, although many studies have been carried out on decellularized esophageal matrix for tissue regeneration and some cancer models [19,20,21], few studies have been done to investigate whether the porcine-derived decellularized esophageal ECM is able to support the attachment and growth of human esophageal squamous carcinoma and primary esophageal fibroblast for potential applications in an esophageal cancer model, as these two cell types are critical to tumor formation and migration [22,23]. Human primary esophageal fibroblast cells (FBCs) are commonly found in the lamina propria and submucosa of the esophagus [24,25]. Studies have also shown that it is important to study the role of FBCs when studying esophageal tumor behavior in the DEM [26]. To study whether the decellularized matrix can support the growth of these two cells, they were cocultured on the DEM. The expression of a secreted extracellular matrix protein, periostin, was observed on the matrix. Periostin has been shown to regulate key aspects of cancer cell behaviors, including cell proliferation, migration, and ECM remodeling [27]. Increased periostin expression is subsequently associated with increased progression of the disease.

In this study, we modified a decellularization method to produce a porcine DEM. The decellularized matrix was fully characterized and the potential to support healthy cells and cancer cells on the matrix was preliminarily examined.

## 2. Materials and Methods

### 2.1. Materials

Adult porcine esophagi were donated from Mary’s Ranch, a USDA-certified slaughterhouse (Pembroke Pines, FL, USA). The decellularizing agents Triton X-100, ammonium hydroxide, trypsin, EDTA, DNase I, RNase, phosphate buffered saline (PBS), sodium deoxycholate (SD), ethylenediaminetetraacetic acid (EDTA), and dimethyl sulfoxide (DMSO) were purchased from Fisher Scientific (Waltham, MA, USA). An antibiotic/antimycotic (AA) containing penicillin, streptomycin and amphotericin-B was purchased from Gibco (Carlsbad, CA, USA). A human esophageal squamous cell carcinoma cell line (KYSE30) was purchased from Sigma-Aldrich (St. Louis, MO, USA). A human primary esophageal fibroblast cells and related culture medium were obtained from Cell Biologics Inc (Chicago, IL, USA). RPMI 1640 cell culture medium was purchased from Gibco (Carlsbad, CA, USA). Primary antibodies collagen type 1, fibronectin, laminin, and periostin were purchased from Abcam (Cambridge, MA, USA). Immunohistochemistry reagents were purchased from Vector Laboratories (Burlingame, CA, USA). The Quant-iT Pico-Green dsDNA Assay Kit was purchased from Thermo Fisher Science (Waltham, MA, USA).

### 2.2. Decellularization of Esophagus

Porcine esophagi were obtained from a local slaughterhouse. Slaughterhouse employees excised the entire digestive tract. The esophagus was then removed inferiorly to the epiglottis and superior to the cardiac sphincter. The length of the total harvested esophagus varied from 12 cm to 20 cm. Samples were rinsed in 5% AA/PBS to remove excess debris and placed in fresh 5% AA/PBS for transportation to the laboratory for further processing [28,29]. At the laboratory, harvested esophagus samples were rinsed in fresh 5% AA/PBS, and each sample was measured, weighed, and characterized. External muscle and adventitia were separated from the submucosa by making a continuous cut along the length of the esophagus with a scalpel. When the external muscle layer was removed, the sample was rinsed with 5% AA/PBS to remove any loose debris.

Native esophagus (NE) samples were placed in a 50 mL collection tube containing 25 mL of 1% Triton X-100 and submitted to 1 h −80 °C freeze, 1 h 37 °C thaw, and 5 min room temperature (RT), and then sonication for three cycles. The Triton X-100 was removed and the sample was rinsed with deionized water (DI water) three times. The sample was submerged in 25 mL of 12 N ammonium hydroxide and placed on a rocker for 10 min. The sample was rinsed with DI water three times. Trypsin/EDTA was then added to the collection tube and placed in the CO_2_ chamber for 10 min at 37 °C. The sample was washed with DI water and kept in place overnight at 4 °C. Post overnight DI water treatment, the epithelial layer was easily detached from the basement membrane and removed from the lamina propria. The sample was rinsed in DI water for three cycles to remove any loose debris. The samples were cut into approximately 0.5 cm^2^ tubular sections, placed in a fresh 50 mL collection tube and treated with 150 IU/mL DNase I and 100 µg/mL RNase I diluted in 20 mL DI water, and then the samples were placed on a rocker for 12 h at 37 °C. The samples were then rinsed with DI water three times. The samples were then treated by 4% sodium deoxycholate placed on the rocker for 4 h at RT. The decellularized samples were rinsed with DI water to ensure removal of any remaining reagent. Decellularized samples in the collection tube were sterilized by rinsing the samples with 70% ethanol at RT. Samples were then rinsed with sterile PBS. Sterile samples can be stored at 4 °C for approximately 2–4 weeks, or at -80 °C for longer-term storage until ready for use.

### 2.3. Characterization of DEM

#### 2.3.1. Removal of Porcine Cells

Decellularized esophageal matrix (DEM) and native esophagus (NE) were placed in 4% paraformaldehyde for 15 min. Graded tissue dehydration and a paraffin embedding process were performed based on our previously established protocol. Paraffin blocks were prepared and 5 μm slides were cut and mounted onto positively charged microscope slides and left to dry for 24 h. Slides were subsequently stained, cover-slipped, and imaged. DAPI stain (5 µg/mL) was applied to separate mounted slides to visually confirm the removal of DNA. Hematoxylin and eosin (H&E) (Sigma Aldrich) staining was used to compare NE and DEM tissue samples to confirm the removal of the cell nucleus.

#### 2.3.2. Quantifying DNA Removal from Native ECM

The Quant-iT Pico-Green dsDNA Assay kit was used to quantify the removal of dsDNA from the NE. Triplicate samples of 0.5 g DEM and their paired 0.5 g NE samples had the DNA lysed from matrix by submerging the samples in 0.2% Triton X-100 and subjecting them to three consecutive freeze/thaw cycles. After sonication, the suspension was aspirated and centrifuged. Dilutions of DNA stock were prepared in order to establish both an upper and lower standard curve. Then, 50 µL of standard curve dilutions, DNA lysate samples, and 0.2% Triton X-100 were added into the wells of a 96-well plate. A total of 50 µL of Pico-Green was added to each well. The plate was protected from the light until it was read at 480/520 nm (excitation/emission) on a fluorescence spectrophotometer (Biotek, Flx800, USA). The amount of dsDNA was calculated by comparing the standard curves of the known dsDNA sample according to the manufacturer’s instructions.

### 2.4. Characterization of Physical Properties of DEM

#### 2.4.1. Observation of DEM Surface Topography

In order to visualize that the physical basement membrane barrier remained intact post-decellularization, NE and DEM tissue samples were fixed in 2.5% glutaraldehyde and subjected to graded dehydration through a serial of ethanol solutions from 70% to 100%. A Leica EM CPD300 critical point dryer (Wetzlar, DE, USA) removed the ethanol and prepared the samples for a 10-µm-thick gold/palladium sputter coat. The surface morphologies were observed under a scanning electronic microscope (SEM, JEOL, JCM-6000Plus).

#### 2.4.2. Observation of 3D Submucosal Microstructural System

NE and DEM tissue were fixed in 10% formalin for 24 h at RT. Samples were then placed in 10% Lugol’s iodine solution for 48 h at RT. Samples were removed from Lugol’s iodine and placed in a 1 mL collection tube surrounded by a loose packing of gauze. Samples were placed in a Bruker Skyscan 1173 MicroCT scanner (Billerica, MA, USA) positioned vertically. Slides were captured in 6 µm segments with a maximum histological output of 0.07. Images were stacked and compiled using the CTvox software (v 3.3.0), (Bruker, Billerica, MA, USA). Post-imaging, the DEM was placed in 10% formalin for 7 days to remove the iodine solution.

#### 2.4.3. Mechanical Strength of DEM Tissue

A flat 0.5 cm × 2.0 cm strip of DEM esophagus and its paired NE tissue were cut and mounted into an Instron E1000 Materials Testing System (Norwood, MA, USA) with a 50 N load cell. Strips were loaded between two stainless steel clamps such that the middle 1.0 cm was the treatment area. Dimensions were measured (length and width) of each sample with calipers to calculate the area. During each tensile test, samples were stretched at a strain rate of 2.0 mm/s until failure or sample protection mode engaged. We converted load-displacement data into an engineering stress–strain curve, then max load, Young’s modulus, and toughness were calculated.

#### 2.4.4. Constituent Component Retention

In order to characterize the retention of native components in DEM, immunohistochemistry was used to confirm the presence of collagen type I, fibronectin, and laminin after the decellularization process. Paraffin-embedded native and DEM samples were cut into 5 µm sections and mounted onto positively charged slides. Sections were submerged in sodium citrate buffer at 95–100 °C for 20 min and placed at RT to cool for 20 min. Slides were blocked with Blocking Solution (Vectashield) and permeabilized with Tween 20-PBS. Primary antibodies collagen type I (1:500, abcam), fibronectin (1:500, abcam), and laminin (1:300, abcam) antibodies were incubated with the samples overnight at 4 °C. Samples were then rinsed and incubated in secondary anti-biotinylated binding IgG at RT for 30 min. Samples were rinsed in Tween-20/PBS and incubated in working ABC solution (Vector Lab) for 5 min at RT. Samples were rinsed and incubated with working DAB solution (Vector lab) for a few minutes until sample stained. Samples were then counterstained with hematoxylin and preserved by adding Permount mounting medium (Fisher Scientific) and a coverslip. Samples were then observed using a Nikon Eclipse TE2000 Inverted confocal microscope.

### 2.5. Characterization of Biological Properties of DEM

#### 2.5.1. ESCC and FBC Growth

KYSE30 cells were cultured in RPMI-1640 media with 10% fetal bovine serum supplemented with 0.5% ampicillin and 0.5% streptomycin in 5% CO_2_ at 37 °C. The esophageal primary fibroblast cells were grown in DMEM culture media supplemented with 10% fetal bovine serum and incubated at 37 °C with 5% CO_2_.

In order to characterize the ability of the DEM to act as a viable scaffold for the study of human cell attachment, we performed an MTT assay on DEM samples to confirm viability of human KYSE30 cells to be metabolically active. A total of 5.0 × 10^5^ KYSE30 cells were seeded onto DEM samples and 24-well plate wells as control. Cell culture media were replaced every 3 days. At each time point, the cell culture medium was removed from the wells and MTT (0.1 mg/mL) solution was added into each well. Samples were placed in the CO_2_ chamber at 37 °C for 4 h. The medium was removed from each well, and DMSO was added to each well. Afterwards, the solution was added into triplicate wells of a 96-well plate and were read at 490 nm.

Additionally, to confirm cell viability and characterize the ability of KYSE30 cells to attach to the DEM, 5.0 × 10^5^ KYSE30 cells were seeded onto DEM and live/dead staining (Thermo Fisher) was used to visualize cell morphology at 1 and 3 days. To observe the cell morphology of primary fibroblast cells, 5.0 × 10^5^ human esophageal primary fibroblast cells (FBCs) were seeded on the DEM, and live/dead staining (Thermo Fisher) was used to stain the cells on the matrix. To further investigate whether cells can infiltrate in the matrix through the ducts or vessels in the matrix, 5.0 × 10^5^ KYSE30 cells were seeded. HE staining was conducted on the cell/matrix after 14 days.

#### 2.5.2. Cell Secreted Proteins on DEM

Studies showed that the upregulation of periostin is known to bind to integrins on cancer cells [30,31], leading to increased cell survival, invasion, angiogenesis, metastasis, and epithelial–mesenchymal transition [32]. For the monoculture groups, 5.0 × 10^4^ KYSE30 and 5.0 × 10^4^ FBCs were seeded in triplicate DEM samples. For the coculture group, 2.5 × 10^4^ KYSE30 cells and 2.5 × 10^4^ FBCs were seeded in triplicate DEM samples. All groups were cultured with 3 mL of 1:1 RPMI 1640: DMEM cell culture media. The cell medium was prepared with 10% fetal bovine serum (FBS) (Gibco) + 1% penicillin–streptomycin–glutamine (PSG) (Thermo Fisher). The cell culture media had no additional growth factors or cytokines added. Time points of 7 and 14 days were established to allow for cell adhesion behavior to initiate. Cell culture media were replaced every 3 days. To observe the periostin expression of cells on the DEM, samples were fixed in 4% paraformaldehyde for 15 min. Samples were rinsed in PBS and then blocked using 5% BSA/PBS for 60 min at RT. Samples were rinsed and incubated with polyclonal periostin primary antibody (1:100, abcam ab14041) and were subsequently DAB stained and observed through Nikon Eclipse TE2000 fluorescent microscope.

In order to quantify the relative expression of perisotin among groups, FIJI ImageJ (NIH) was used. Previously DAB-stained slide images were deconvoluted in FIJI and converted into 8-bit gray scale images. Images were analyzed in triplicate measuring mean gray scale for identically sized areas of matrix. Each group was tested in triplicate, and analyzed using both one-way ANOVA and two-way ANOVA using Tukey’s post-hoc multiple comparisons test.

### 2.6. Statistical Analysis

All experiments were performed in triplicate. GraphPad Prism v9.0 (GraphPad, San Diego, CA) was used for ANOVA tests to measure statistical significance. A Tukey test was carried out for multiple comparisons between groups. The difference was considered to be statistically significant when *p* < 0.05. Whenever ANOVA indicated a significant effect, we carried out pairwise comparison of means using the Newman–Keuls test. The assumptions of the analysis of variance of residuals and normal distribution were verified. The sample sizes (*n*) are indicated in the figure legends.

## 3. Results

### 3.1. DEM Characterization

**Figure 1:**
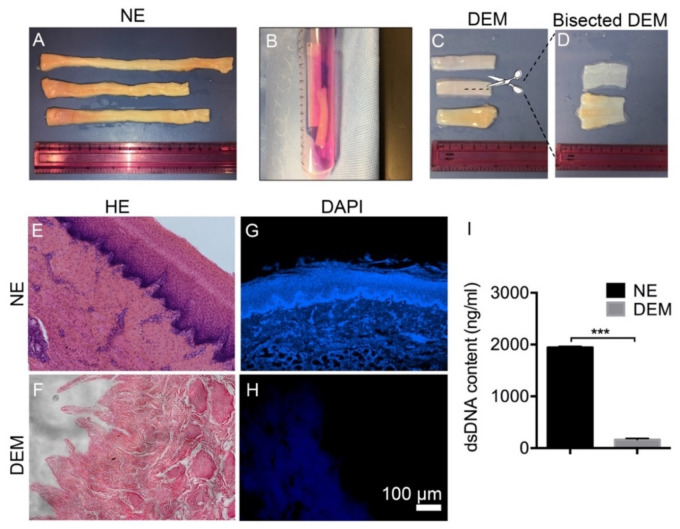
The removal of cell nuclei of pig esophagus: (**A**) Macroscopic image of freshly harvested native esophagi. (**B**) During decellularization, esophagus in Trypsin/EDTA. (**C**) Post-decellularization, DEM tissue has a pale white appearance. (**D**) DEM transversely cut exposes the lumen and the basement membrane of the tissue. H&E staining shows the presence of porcine cells and nuclei on native esophagus (NE) (**E**) but they disappeared on decellularized esophageal matrix (DEM) (**F**). DAPI staining on NE (**G**) and DEM (**H**). Pico-Green assay shows 92% dsDNA was removed (*n* = 3) *** (*p* < 0.0001) (**I**).

The purpose of this study was to determine the effectiveness of the decellularization process while maintaining the native microstructure of the extracellular matrix and also to examine whether the decellularized matrix can support cell growth. Porcine esophagus (Figure 1A) was cut into small pieces and put in a tube for decellularization (Figure 1B). Post-decellularization, the tissue appeared milky-white with some translucency. The macroscopic anatomical view of the esophageal tissue grossly shows that the esophageal tissue, originally a red/yellow flesh color, became a pale white color following decellularization (Figure 1C,D). Other than that, the DEM maintained its general structure following the decellularization process. Bisecting the DEM uncovered no visually observable damage to the basement membrane or submucosa following the decellularization process. Because collagen has a high affinity for water, freshly treated DEM tissue (saturated with water) has a slippery texture and is difficult to handle and cut. To observe the removal of porcine cells from the native tissue, H&E staining was performed and visually confirmed the presence of nucleated stratified squamous cells of the mucosal layer, dermal papillae, and stromal cells of the submucosa in the NE (Figure 1E). After decellularization, Figure 1F shows the complete removal of the stratified squamous layer of the esophageal mucosa and characterized the preservation of the dermal papillae along the basement membrane of the native esophageal tissue. DAPI staining confirmed the presence of native DNA in both mucosal and submucosal layers (Figure 1G). DAPI confirmed the removal of cell nuclei from the DEM (Figure 1H). The Pico-Green dsDNA assay showed that DNA removal through the decellularization protocol consistently removed approximately 92.5% of the quantifiable native porcine double-stranded DNA (*n* = 3) (*p* < 0.0001) (Figure 1I).

**Figure 2:**
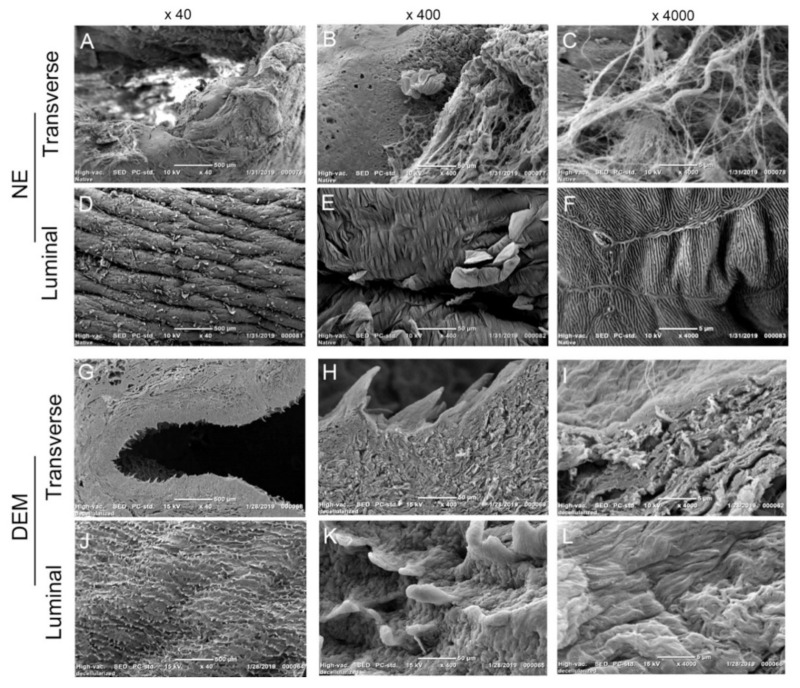
SEM imaging of NE and DEM. The transverse SEM imaging of NE shows mucosa and submucosal layers at different magnifications (**A**–**C**), and the lumen side at different magnifications (**D**–**F**). SEM imaging of the transverse DEM at 40×, 400×,4000× (**G**–**I**) and the DEM lumen surface at 40×, 400×, 4000× (**J**–**L**).

In order to characterize the morphologies of the DEM matrix, SEM imaging was used (Figure 2). Cross-sectional SEM imaging of the NE visualizes both the mucosal and submucosal layers of the tissue at 40x (Figure 2A). Upon closer inspection of the NE, the basement membrane and stratified epithelial layer can be visualized (Figure 2B). Bundles of fibers can be seen at 4000x (Figure 2C). SEM imaging of the lumen surface of the NE shows the stratified epithelium completely covers the basement membrane. It is noteworthy that there appears to be dead layers of cells sloughing off and the presence of many folds on the inter-luminary epithelial surface (Figure 2D). Closer inspection of the NE lumen visualizes the folds of the surface and sloughing layers (Figure 2E). An interesting observation can be seen at 4000x, where the adhering junctions between the epithelial cells can be visualized as well as a fingerprint-like pattern along the lumen surface of the NE (Figure 2F). After decellularization, the SEM imaging of the DEM grossly visualizes the removal of the stratified epithelium (Figure 2G) of the mucosal layer. Closer inspection of the DEM showed the preserved basal papillae observed at 400x (Figure 2H). The basal papillae measure between 25–100 µm tall and appear to be formed of interwoven fibers (Figure 2I). The lumen surface of the DEM showed distributed basal papillae throughout the basement membrane (Figure 2J). The papillae appear to have a row-like orientation along the whole basement membrane. The row-like organization of the papillae appears to form a depression in the basement membrane that possesses a channel-like morphology (Figure 2K). Upon closer examination at 400x, the groove-like pattern was clearly shown (Figure 2L).

**Figure 3:**
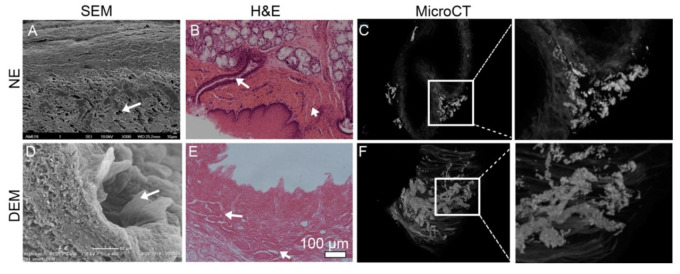
Images show the pores and ducts of the esophageal matrix. SEM images show the pores in NE (**A**) and their existence in DEM (**D**) (arrows). H&E staining can clearly show the pores underlying vasculature or ducts in NE (**B**) and in DEM (**E**). MicroCT imaging of NE tissue shows vessels throughout the submucosa (**C**). Continuous vascular networks from the white box of C can be visualized as small as 6µm in diameter. (**F**) MicroCT imaging of the DEM tissue indicates preservation of the complex vascular network throughout the submucosa. Large cavernous spaces and independent tubular networks can be visualized in the box in (**F**).

When the epithelium layer with epithelial cells was removed and the basement membrane was exposed, the pore opening to the lumen-side surface could be clearly visualized. To further observe pores and ducts in the matrix, SEM, HE staining, and microCT imaging were performed. In the NE, the pores are grossly surrounded by a ring of the basal papillae, and have a patent duct leading to the lamina propria. SEM further showed the pores that do not appear uniformly distributed (Figure 3A), but observationally appear more commonly on the DEM tissue collected closer to the cardiac region of the esophagus (Figure 3D). These pore features were confirmed by cross-sectional histology of the NE tissues (Figure 3B) and DEM (Figure 3E). Elucidation of the ductal system below the surface is limited by SEM and histology. MicroCT imaging was then employed to better characterize vessels below the surface. MicroCT imaging showed clusters of iodine-filled networks throughout the lamina propria and submucosa. In the NE tissue, the vessels can be seen clustered in the thicker folded areas of the tissue (Figure 3C). Although imaging does not describe if vessels are vascular, lymphatic, or ductal networks, the microCT imaging of the thick region of the DEM shows the preservation of these complex vessel systems in the submucosa (Figure 3F). In comparing the native and DEM groups, the vessels appeared well preserved post-decellularization. These ducts and vessels may have roles in regulating cell infiltration or migration.

### 3.2. DEM Architecture and Mechanical Properties

**Figure 4:**
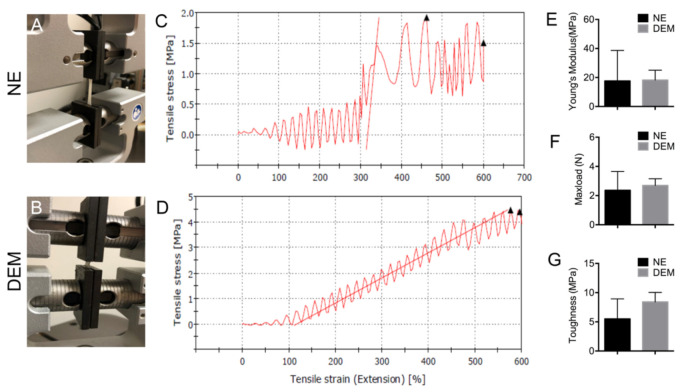
The mechanical properties of NE and DEM. (**A**) NE and (**B**) DEM mounted on the Instron structural testing machine. A representative mechanical stress–strain curve for NE (**C**) and DEM (**D**). (**E**) Young’s modulus of NE and DEM (*n* = 9) (*p* = 0.23). (**F**) Max load of NE and DEM (*n* = 9) (*p* = 0.11). (**G**) The stiffness of NE and DEM (*n* = 9) (*p* = 0.07).

To further study how well DEM tissue retained the mechanical properties of the native porcine esophageal ECM, max load, Young’s modulus, and toughness were calculated using the Instron E1000 (Figure 4A,B). The NE was completely devoid of any decellularization treatment and as a result the NE contained both the tightly locking stratified epithelial layer and submucosal layer (Figure 2A–F). The DEM sample was fully decellularized and subsequently was only composed of the remaining submucosal layer (Figure 2G–L).

Both NE and DEM samples were tested to 600% tensile extension (Figure 4C,D). The native tissue exhibited an extension stress maximum between 1.5–2.0 MPa at around 450% extension, while the DEM tissue expressed an extension stress maximum between 4.0 MPa and 4.5 MPa at 600% extension. The slope of the stress–strain curve was used to calculate the Young’s modulus. No samples physically failed during testing and all completed to the 2.5 cm limit, subsequently triggering instrument sample protection mode. Then, *t*-tests were performed on the data comparing the native and DEM groups. There was no significant loss of max load (*p* = 0.11), toughness (*p* = 0.07), or Young’s modulus (stiffness) (*p* = 0.23) as a result of the decellularization process (Figure 4E–G). Through multiple tests there was significant variation in the NE stress–strain curve due largely to the stratified squamous epithelial (mucosal) layer tearing at approximately 300% extension.

**Figure 5:**
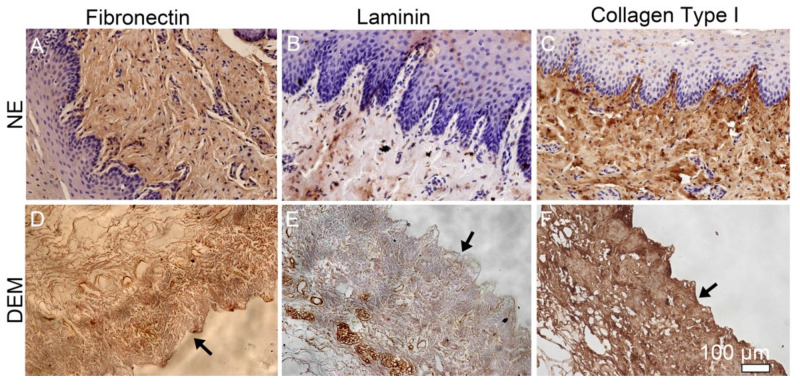
The immunohistochemical staining of three components of matrix: fibronectin, laminin, and collagen type I on NE (**A**–**C**) and DEM (**D**–**F**) (black arrows show the luminal surface).

In order to confirm the retention of specific constituent components of the ECM through the decellularization process, immunohistochemistry was used to characterize the presence of collagen type I, fibronectin, and laminin in the DEM. NE stained positive for fibronectin throughout the matrix (Figure 5A). Laminin can be seen in the NE along the basement membrane and lining vessels within the submucosa (Figure 5B). NE visibly expresses collagen type I throughout the lamina propria and submucosa (Figure 5C). In DEM, fibronectin can be observed, but certainly to a lesser extent than in the NE (Figure 5D). DEM stained positive for laminin that appeared specifically in the areas where pores or ducts exist (Figure 5E). Similar to NE, DEM stained positive for collagen type I consistently throughout the matrix (Figure 5F).

### 3.3. Cell Viability and Morphology on DEM

**Figure 6:**
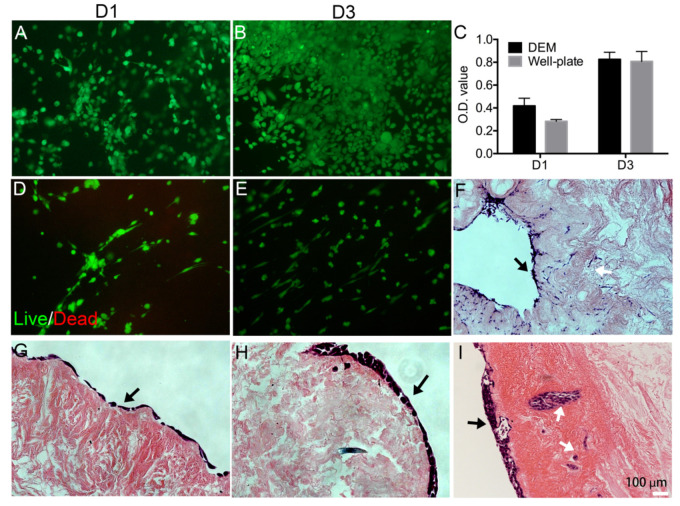
Cell viability on DEM. Live/dead staining of human KYSE30 cells on DEM after 1 day (**A**) and 3 days (**B**). MTT result reflects cell proliferation on DEM related to well plate (**C**). Live/dead staining of human primary esophageal fibroblast cells on DEM after 1 day (**D**) and 3 days (**E**). H&E staining confirmed that human primary esophageal fibroblast cells on the DEM can further prove the presence of such pores and ducts, as FBC moving through the lamina propria (**F**) (white arrows show the pores and ducts in which cells migrated). H&E staining shows that the attachment of KYSE30 cells on the lumen side of the matrix on 1 day (**G**) and 3 days (**H**); after 14 days the KYSE30 migrate into the DEM through the pores (white arrows) (**I**) (black arrows show the luminal surface).

In order to determine the ability of the porcine-derived DEM to host human KYSE30 cancer cells, live/dead staining and MTT assay were used. Live/dead staining visualizes cell viability and proliferation. Day 1 imaging shows that there was a high seeding efficiency on the DEM with very few dead cells (Figure 6A). Continuing in time, live/dead staining images showed an increase in cell density in the DEM on day 3 (Figure 6B). At the end of the experiment, the entire sample was covered with live KYSE30 cells. MTT results showed that there was no significant difference in cell proliferation between the DEM and the well plate on day 1 or day 3 (*p*_day 1_ = 0.611), (*p*_day 3_ = 0.999) (Figure 6C). Similarly, live/dead staining visualizes cell morphology of primary esophageal fibroblast cells. Live/dead staining images showed a slight increase in cell density of fibroblast in the DEM with time (Figure 6D,E).

Cross-sectional H&E histology shows a single layering of ESCC cells on the DEM prolific along the lumen surface (Figure 6G). As time increased, cells began the process of stratification (Figure 6H). The DEM illustrates the functionality to host human cancer cells over time for culture. Additionally, H&E staining of human fibroblast cells seeded onto the lumen surface attach and are viable after 14 days (Figure 6F). Cross-sectional histology visualizes the ability of the fibroblasts to attach within the submucosa and infiltrate into the matrix using these pores and vessels (Figure 3F). H&E staining also showed KYSE30 cells had filled in the pores in the basal papilla by day 14 (Figure 6I). It seems that cells infiltrated into the pores through ducts. This is an interesting phenomenon that was observed in the cell behavior. More experiments should be further conducted to prove these observations.

### 3.4. Periostin Expression and Deposition

**Figure 7:**
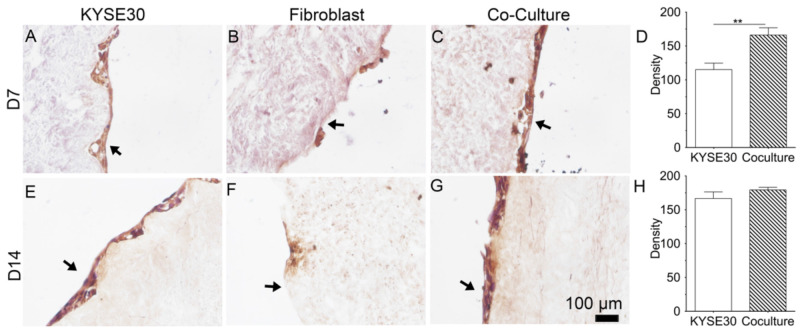
Immunochemical staining of periostin on KYSE30(**A**,**E**), fibroblast (**B**,**F**), and their coculture (**C**,**G**) at day 7 and 14. FIJI quantification of the positive periostin staining based on 8-bit gray scale images (**D**,**H**) (*n* = 3) (black arrows show the luminal surface). ** *p* < 0.001.

To investigate whether coculture of ESCC and FBC can stimulate the expression of periostin protein, immunohistochemical staining was used to study the deposition of periostin onto the DEM. After day 7, KYSE30 shows the expression of periostin on DEM (Figure 7A). FBCs expressed few periostin on the matrix (Figure 7B). When cells were cocultured, the expression of periostin relative to the KYSE30 alone group became visibly clearer (Figure 7C). The presence of stromal fibroblasts stimulated the periostin expression of KYSE30 on the DEM. The images were visually characterized using microscopy and quantitively analyzed using FIJI ImageJ v2.0.7 software. The density of the positive staining of periostin in the coculture group is significantly higher than that of monoculture of KYSE30 group (Figure 7D). With the time increasing to day 14, KYSE30 showed an increase in periostin expression relative to the day 7 (Figure 7E), but FBC cells showed no change or diminishing in expression on the matrix at day 14 (Figure 7F). In the coculture group, cells continued to express elevated periostin levels relative to the day 7 cocultured group on DEM and the KYSE30 alone group (Figure 7G). The density of the positive staining of periostin in the coculture group was slightly higher than that of the monoculture KYSE30 group but there was no significant difference (Figure 7H). Compared to the absolute density of the staining at day 7, the staining density of monocultured and cocultured KYSE30 increased at day 14.

## 4. Discussion

Decellularized esophageal extracellular matrix has been widely studied for the purposes of tissue regeneration and surgical implantation. However, there has been little previous research to use the native derived and heterogeneously constructed DEM for potential application in cell growth of esophageal cancer and esophageal primary fibroblast cells. The key issue for studying cancer cell adhesion into the submucosa is the ability to accurately recreate the physical and biological cues that regulate cell attachment on the ECM. There have been many attempts to construct a 3D extracellular matrix [33,34,35]. Nocera showed that even high-porosity 3D-printed scaffolds could not model cell migration into the matrix, indicating that all models embody some limitation in design [34]. However, the efforts to construct a mimetic 3D-ECM scaffold is primarily limited due to the difficulty in recreating the native duct network of the submucosa of heterogenous ECM. In this study, we used a modified decellularization protocol to decellularize porcine esophagus. This modified method preserved the microstructure in the base membrane. We used this DEM scaffold to study the attachment of human esophageal squamous cell carcinoma and primary esophageal fibroblast cell. We found that the DEM scaffolds were able to host KYSE30 cells and fibroblast cells to grow on the matrix and also found that cells infiltrated into the microducts or pores. Microscopy also characterized the preservation of the basal papillae and the denser lamina propria (Figure 2). These data implied that the decellularization process did not significantly impair the physical microstructure of the matrix. The pores along the basement membrane of the DEM and the ducts and vessels that network throughout the submucosa (Figure 3) not only aid in supplying nutrients through the matrix, but also add to the physical mechanisms that may facilitate cell infiltration [15].

Further, the results of material testing indicate that there was no significant loss of matrix strength, max load, or stiffness resulting from the decellularization process (Figure 4). It is noteworthy to consider that once the tightly bound epithelial cells of the mucosal layer began failing at approximately 300% stress, the NE samples expressed a steep curve and began to display similar mechanical properties as DEM before the test concluded. These data indicated that although the mucosal layer complicated the analysis of mechanical properties of ECM, the decellularization process did not significantly damage the mechanical properties of the esophageal matrix at peak strain.

The creation of the DEM addresses complex structural challenges and is also shown to retain the native constituent components collagen, fibronectin, and laminin. In this study, we mainly observed these three components in the matrix as they are critical components in maintaining the matrix mechanical properties as well as modulating the cell activity. IHC of the NE and DEM both expressed collagen, fibronectin, and laminin throughout the matrix. Visually, the expression of these components appeared diminished in the DEM, while the retained amount of these components was not quantified. In this study, we used mild conditions and chemicals to gently decellularize the esophagus. The IHC staining showed that these matrix components were preserved. They are important for cell migration signaling [11,12,30] and therefore the preservation has value.

Cancer cell and fibroblast attachment behavior when seeded onto the DEM is of vital importance to accurately study cellular activity. For example, it has been previously described that the density of the basement membrane predominantly prevents epithelial cancer cells from easily invading into the submucosa [36]. The DEM scaffold is also able to interact with cancer cells while acting as a physical barrier and supporting cell adhesion, which is a positive in creating a biomimetic environment. It is of value that we were able to characterize that the DEM scaffold exhibited tracks of cancer cells within the ductal pathways of the DEM (Figure 3 and Figure 6). These cells appear to be associated with the attachment to the areas proximal to basement membrane pores and submucosal tracts. The mechanism remains unknown.

In this study, we examined whether the coculture of fibroblast can promote the secretion of a cellular protein, periostin. Using immunohistochemistry, we observed that the periostin staining appeared to increase over time. This result implied that the matrix can support periostin secretion of cancer cells and also that fibroblasts promoted the expression of cancer cellular periostin. It would be a key finding in the future to quantify the retention of perisotin by the DEM and to discover the mechanism by which the matrix regulated periostin secretion. There should be further studies on this. Preliminarily, the DEM did appear successful in maintaining this quality, and therefore made DEM a quality biomimetic matrix for cancer study.

The development of a porcine-derived DEM, which maintains the physical and biological characteristics of the native ECM, and provides the relevant cues to more accurately study cellular activities seen in human esophageal cancer, is significantly encouraging to better understand the cellular cues that drive cancer cell attachment. However, there are limitations to the current study. First, quantification of fibronectin, laminin, and other native glycoproteins was absent from this study. Second, whether cell adhesion is mechanically or biologically driven remains to be discovered. Third, the tumor markers and cell–cell interactions were not thoroughly investigated for understanding cancer cell behavior and the effect of fibroblasts in the matrix. Future experiments might focus on the role of DEM in cellular pathway activation and regulation of tumor development. Even with these limitations, this DEM may bring new potential to support these future studies and create an artificial esophageal tumor tissue in vitro.

## 5. Conclusions

This study found that the native porcine esophagus was successfully decellularized while maintaining native collagen, fibronectin, and laminin to a lesser extent within the native heterogenous layering. The DEM also maintained the native surface pores and submucosal vessel network. The DEM was an effective scaffold for the ex vivo study of cancer cell attachment. The presence of retained heterogenous architecture and duct networks provides the potential for greater understanding of how anatomy can facilitate cancer cell movement. Ultimately, the recapitulation of the native ECM properties in the DEM scaffold holds the potential to provide a matrix microenvironment for creating a biomimetic tumor model in the future.

## Data Availability

The data presented in this study are available on request from the corresponding author.
